# Therapeutic effect of acute and chronic use of different doses of vitamin D3 on seizure responses and cognitive impairments induced by pentylenetetrazole in immature male rats

**DOI:** 10.22038/IJBMS.2021.60123.13328

**Published:** 2022-01

**Authors:** Hong Jiang, Suying Zhang

**Affiliations:** 1 Department of Pediatric, Weinan Maternal and Child Health Hospital, Weinan, 714000, China; 2 Department of Child Health, Weinan Central Hospital, Weinan, 714000, China

**Keywords:** Apoptosis, Epilepsy, GABA receptor, Inflammation, NMDA receptor, Vitamin D3

## Abstract

**Objective(s):**

This study aimed to evaluate the effects of acute and chronic intake of different doses of vitamin D3 on seizure responses and cognitive impairment induced by pentylenetetrazole (PTZ) in immature male rats.

**Materials and Methods:**

Sixty-six immature male NMRI rats were divided into control (10), epileptic (10), and treatment groups (46). The stage 5 latency (S5L) and stage 5 duration (S5D) were assessed along with the shuttle box test. Levels of antioxidant enzymes and inflammatory factors along with genes involved in inflammation, oxidative damage, apoptosis, and mTORc1 were measured in the hippocampus tissue of the brain of controlled and treated rats. Serum levels of parathyroid hormone (PTH), vitamin D, calcium, and phosphorus were also assessed.

**Results:**

The results showed that the ability to learn, memory consolidation, and memory retention in epileptic rats were reduced. In addition, S5D increased and S5L decreased in epileptic rats, while being effectively ameliorated by chronic and acute vitamin D intake. The results showed that vitamin D in different doses acutely and chronically decreased the levels of oxidative and inflammatory biomarkers in hippocampus tissue and inhibited the expression of genes involved in inflammation, oxidative damage, apoptosis, and mTORc1 in the hippocampus tissue of epileptic rats.

**Conclusion:**

The results showed that vitamin D in different doses acutely and chronically could improve cognitive impairments and convulsive responses in epileptic rats by improving neurotransmission, inflammation, apoptosis, and oxidative damage.

## Introduction

Epilepsy is a chronic non-communicable brain disease that can affect people of any age (1). Around 65 million people worldwide suffer from epilepsy. The prevalence of premature death in people with epilepsy is up to three times higher than in the general population. Nearly 80% of people with epilepsy live in low- and middle-income countries and do not get the treatment they need. In high-income countries 49 per 100000 people and in low- and middle-income countries 139 per 100000 people are diagnosed with epilepsy every year (2, 3). Anti-seizure medications are the mainstay of epilepsy treatment. However, the drugs have various effects and adverse effect profiles such as cognitive impairments (4). Therefore, it is important to know the mechanisms involved in epilepsy and use new treatment strategies to prevent and treat this disease.

Seizures as the manifestation of epilepsy are the result of excessive electrical discharge in a group of cells in different parts of the brain. Many of the mechanisms involved in causing seizures are known, however, in about 50% of cases worldwide the cause of the disease is still unknown (5). Neuroinflammation, apoptosis, and oxidative damage along with glutamate-mediated excitotoxicity and inhibition of the gamma-aminobutyric acid (GABA) pathway are common underlying events in neuronal degradation and are involved in the pathogenesis of epilepsy (6, 7). Neuroinflammation, apoptosis, and oxidative stress reinforce each other and lead to nerve damage by altering the cellular signaling involved in the production and dissemination of action potentials, which are the most important factors in epilepsy and cognitive impairment (7). Therefore, it is hypothesized that the use of anti-inflammatory drugs and anti-oxidants can inhibit the clinical onset of symptomatic epilepsy and progression of spontaneous seizures and delay their onset.

Studies have shown that therapeutic effects of anti-inflammatory drugs and anti-oxidants to reduce seizures are achieved by targeting molecular signaling pathways such as IL-1β, TLR4, P2X7, and Nrf2 (8, 9), while their therapeutic effects on COX-2 inhibition to reduce spontaneous seizures are controversial (10). Moreover, inhibition of the mammalian target of rapamycin (mTOR) with rapamycin has anti-epileptogenic potential (11). However, because rapamycin treatment causes unwanted side effects, it is recommended to study rapamycin substitutes as antiepileptic drugs. Studies have shown that neuroinflammation, oxidative damage, apoptosis, and neurotransmission, which are involved in the development of epilepsy, can be due to malnutrition and inadequate intake of certain minerals and vitamins such as vitamin D (12, 13).

Vitamin D, as a neurohormone that regulates the release and function of neurotransmitters and neurotrophins, has anti-oxidant anti-inflammatory properties, and is a neuroprotector. It increases neurotrophic factors such as nerve growth factor which further promotes brain health. Moreover, it is also helpful in the prevention of epilepsy and cognitive impairments (12). However, studies have consistently found that vitamin D levels are significantly low in individuals with epilepsy and cognitive impairments (12, 13). Epileptic seizures have been also reported to show seasonal variation and peak in January, reflecting fluctuations in vitamin D levels (14). In addition, it has been well shown that vitamin D deficiency is effectively associated with cognitive impairments (12) and seizures (12) in the elderly, adults, and children in middle- and low-income countries (15, 16). Given that children as one of the age groups are prone to vitamin D deficiency and it, in turn, can be one of the risk factors for epilepsy and cognitive impairments (12, 13), it is necessary to study this field in order to understand the molecular mechanisms involved in them.

Children are considered an age group that is very prone to vitamin D deficiency. Low plasma levels or deficiency of this vitamin cause disorders of nervous system development, immune system dysfunction, and cognitive disorders. Moreover, children taking anticonvulsants are at risk for vitamin D deficiency (17). It has also been shown that bone mineral density is low in people taking anticonvulsant drugs, and the protective effects of vitamin D3 in correcting it have been observed in patients with epilepsy (1). Moreover, both treatments with antiepileptic drugs and the presence of epilepsy cause cognitive impairments (16) and have a negative effect on serum vitamin D levels (18). These results clearly indicate the need for vitamin D supplementation with antiepileptic drugs. 

Vitamin D deficiency can affect many aspects of brain function and leads to attentional, behavioral problems, and cognitive impairment (12). The rate of immature neuronal branching, differentiation, and protein expression in neurons may be affected by vitamin D deficiency as well as neurotransmission between neurons and neuronal synaptic plasticity (19). Vitamin D deficiency has also been implicated in the pathogenesis of cognitive impairments in Parkinson’s disease and Alzheimer’s disease, dementia (12, 20). It has been well shown that vitamin D deficiency is involved in cognitive impairments in different age groups (12). Several lines of epidemiological evidence together with some experimental data also suggested a beneficial role of vitamin D in epilepsy and cognitive impairments (21, 22). The use of vitamin D3 is also useful in reducing the duration of seizures and delaying their onset in patients with epilepsy. Moreover, the anticonvulsant effect of vitamin D has been demonstrated in electrical hippocampal seizures (23), and PTZ-induced seizures (24). However, despite the many studies that have been done in this field, the effect of vitamin D on the improvement of epilepsy and related cognitive disorders in children is not fully understood. Moreover, due to the role of vitamin D3 in seizures and lack of identification of accurate mechanisms of its action in pathophysiological pathways causing seizures and epilepsy-related cognitive impairments, it is necessary to conduct a study in this field. Therefore, this study aimed to evaluate the effects of acute and chronic intake of different doses of vitamin D3 on epileptic seizures and cognitive impairments induced by PTZ in immature male rats. 

## Materials and Methods

***Animal and ethical statement***

All stages of this study were in accordance with the guidelines of the Helsinki Declaration (Helsinki Declaration, revised, 2013) and were also approved by Weinan Maternal and Child Health Hospital animal ethical committee (approved no. WNFY210428). In this study, sixty-six immature male NMRI rats (100–130 g) were kept in a breeding-house controlled for temperature (22±2 °C), brightness (12 hr bright: 12 hr dark), and humidity (30%).

***Study design***

**Figure 1:**
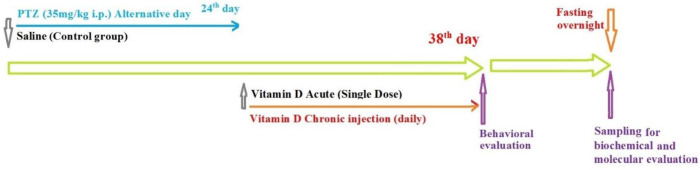
Schematic diagram of research process in this study

In this study, sixty-six immature male NMRI rats were divided into control (10 rats), epilepsy (10 rats), and treatment groups (46 rats). The control group was divided into acute and chronic subgroups and the treatment group was divided into three subgroups: acute (18 rats), chronic (18 rats), and diazepam (10 rats, 2 mg/kg) groups. Acute subgroups received vitamin D3 as a single dose of 50, 100, and 150 μg/ml by intraperitoneal injection (IP), and chronic subgroups received vitamin D3 daily for 2 weeks at doses of 50, 100, and 150 μg/ml. In all groups, except the control group, to induce kindling, PTZ (35 mg/kg body weight) was injected every 48 hr intraperitoneally (Figure 1) (25). 

***Induction and evaluation of epilepsy***

For the epileptic groups, according to the standard procedure, several injections were performed with an interval of 48 hr until the rats were completely kindled (25). On the last day in all groups after injection of 60 mg/kg PTZ, tonic seizure threshold, and mortality rate were recorded. The control group received normal saline during this period. Vitamin D was taken daily after this period for two weeks. After that, the animals’ behaviors were monitored. Animal seizure responses were assessed within 20 min after PTZ injection and were classified as follows according to previous research (25). 

Step zero: No answer

Step 1: Contraction of the muscles of the face and ears

Step 2: The body’s contraction wave

Step 3: Myoclonic jumps and standing on two legs

Step 4: Fall to the side

Step 5: Falling on the back and general tonic and clonic seizures 

**Figure 2:**
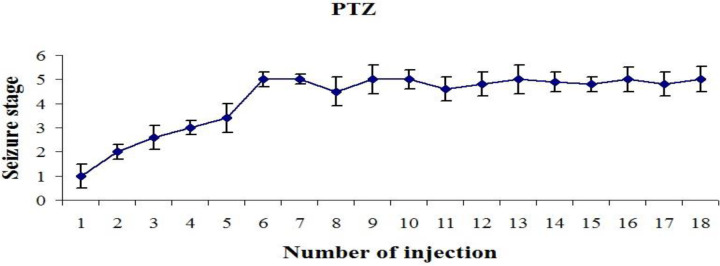
Assessment of convulsive behavior. After each injection, the seizure scores were monitored and scored. PTZ injections significantly increased seizure severity. Each seizure score is shown as the mean ± SEM

After the first injection, some rats showed the first or second stages of seizure, and with continued injections, the seizures gradually progressed in the rats. The injections were continued in each group until each animal showed the fifth stage of seizures twice in a row. By the 8–10th injection, all rats responded with a full seizure. Thus this animal was considered as a kindle animal. The time duration for the animal to reach the fifth stage of seizure (stage 5 latency: S5L) and the duration of time the animal was in the fifth stage of seizures (stage 5 duration: S5D) were measured (Figure 2). 

***Evaluation of memory and learning in controlled and treated rats***

Two weeks after the end of kindling, the animals’ memory and learning activity were assessed by evaluating passive avoidance via shuttle box test (26). The apparatus shuttle box consisted of two chambers (a dark and an illuminated chamber), with a steel grid floor to deliver foot-shocks. The rat was placed in the illuminated compartment and had free access to all parts of the apparatus for 300 sec for habituation. One day after the last PTZ injection, the animal was placed in the illuminated chamber and allowed to explore for 30 sec. After 30 sec, the door between the two chambers was lifted, and thereafter its latency to enter the dark compartment was recorded. The interval between the placement of the rat in the light chamber and its entry into the dark chamber was measured as entrance latency. Immediately, the door was closed once the animal entered the dark compartment, and a single electric foot shock (65 V AC, 50 Hz) for 5 sec was delivered. One day after this learning session, the animal was placed in the illuminated chamber and the latency to re-enter the dark chamber was measured. This was known as consolidation memory. The same procedure was used for the long-term memory test which was performed three days after the learning/training session. Foot shocks were not administered during the retention tests. Rats that did not enter the dark compartment during the given 5-min period were removed from the apparatus and the latency was considered to be 300 sec.

***Sampling and tissue preparation***

After the end of the treatment period, the animals were anesthetized with a combination of ketamine (80 mg/kg) and xylazine (8 mg/kg). Blood samples were taken from the hearts of the rats and the brain was removed after the rats were sacrificed. The hippocampus tissue of the brain of all rats was removed and then frozen in liquid nitrogen. The hippocampus tissue was manually homogenized in 1 ml of cold phosphate buffer (pH = 7.4, 0.1 M). Afterward, samples were centrifuged at 10000×g for 10 min at 4 °C, and supernatants were used for biochemical assessment. 

***Biochemical assessments***

Serum levels of parathyroid hormone, vitamin D, calcium, and phosphorus were also assessed. For evaluating vitamin D status, serum 25(OH)D3 levels were measured using the radioimmunoassay method (Immunodiagnostic Systems, Boldon, UK), and serum Ca and phosphorus were measured using a photometric test (Shanghai Crystal Day Biotech Co., Shanghai, China). The enzyme-linked immunosorbent assay (ELISA) method was used to measure serum levels of parathyroid hormone (PTH) (Shanghai Crystal Day Biotech Co., Shanghai, China).

***Oxidative and inflammatory biomarkers assessments***

Levels of anti-oxidant enzymes and inflammatory factors were measured in hippocampus tissue. Malondialdehyde (MDA) level, an index of lipid peroxidation, was estimated according to the method of Esterbauer and Cheeseman (1990) (27). The amount of MDA formed was measured by the reaction with thiobarbituric acid using a spectrophotometer. The results were expressed as nmol MDA/mg protein. Glutathione (GSH) level was estimated by the method of Fukuzawa and Tokumurai (1976) (28). This method was based on the reaction of GSH with DTNB formed by the colored product. Results were expressed as μmol GSH/mg protein. The activity of total superoxide dismutase (SOD) was indirectly assayed by inhibition of the pyrogallol autoxidation in a reaction media as previously described (29). The activity of catalase (CAT) was determined by the spectrophotometric method by depletion of H_2_O_2_ and expressed as μmol of H_2_O_2_ consumed/min/mg protein (30). The activity of glutathione peroxidase (GPx) using the Rotruck method was measured and expressed as U/mg protein (31). The protein content was estimated according to the method of Lowry *et al*. (1951).

***Molecular assessments***

Expression of NF-κB, TNF-α, IL-1β, HMGB1, TLR4, COX-2, TGF-β, B-cell lymphoma 2 (BCL-2), Caspase-3, Caspase-9, Nfr2, and mTORc1 was measured in the hippocampus tissues of the brain of controlled and treated rats. Total RNA was isolated using the TRIzol^®^ LS reagent, following the manufacturer’s instructions (Breda, The Netherlands). The concentration and purity of RNA were determined at 260/280 nm using a NanoDrop 2000 spectrophotometer (Thermo Fisher Scientific, Wilmington, DE, USA). Oligo dT primers were used for cDNA synthesis. Real-time PCR reactions were run on a Rotor-Gene 6000 (Corbett Research, Australia), using Real Q Plus 2x Master Mix Green (Ampliqon, Denmark). The sequence of primers used in this study was extracted from previous studies and is given in Table 1. Normalization of the expression of target genes was performed with the expression of the gene GAPDH as a housekeeping gene. The expression of target genes was also calculated using the 2^ΔΔCt^ method (32).

***Statistical analysis***

The results are presented as the mean ± standard error of the mean (SEM). All data were recorded using Statistical Package for Social Sciences (SPSS 19.0). Data were analyzed using one-way analysis of variance (ANOVA) followed by *post hoc* multiple comparisons Tukey’s test to compare the results of different treatment groups. The statistical significance was set at *P*<0.05. 

**Table 1:** The sequence of primers used in this study for the real-time PCR reaction

Gene	Sequence	Size product	Accession no.
HMGB-1	F: 5´-GTAATTTTCCGCGCTTTTGT-3′ R: 5´-TCATCCAGGACTCATGTTCAGT-3′	114	NM_012963.2
TLR-4	F:5´-AGGATGATGCCAGGATGATGTC-3′ R: 5´-TCAGGTCCAGGTTCTTGGTTGAG-3′	195	NM_019178.2
NF-κB	F: 5′-GCACCAAGACCGAAGCAAT-3′ R:5′-CGTAACCGCGTAGTCGAAGA-3′	143	NM_001276711.1
IL-1β	F:5´- AAAAATGCCTCGTGCTGTCT -3′ R: 5´- TCGTTGCTTGTCTCTCCTTG -3′	118	NM_031512.2
TNF-α	F:5'-CTCTTCTGCCTGCTGCACTTTG-3' R: 5'- ATGGGCTACAGGCTTGTCACTC-3'	186	M10988
COX-2	F:5´-CAAGCAGTGGCAAAGGCCTCCA-3′ R: 5´- GGCACTTGCATTGATGGTGGCT-3′	459	NM_017232.3
mTORc1	F:5′- GGTGGACGAGCTCTTTGTCA -3′ R:5′- AGGAGCCCTAACACTCGGAT -3′	225	NM_019906.2
Nrf2	F:5´-AAAGACAAACATTCAAGCCGATTAG-3´ R: 5´-TTGCTCCTTGGACATCATTTCAT-3′	141	NM_031789.2
Bcl2	F: 5′-GCTACGAGTGGGATACTGGAGATGA-3′ R: 5′-ACAGCGGGCGTTCGGTTG-3′	103	NM_016993.2
Caspase-3	F: 5′-GCAGCAGCCTCAAATTGTTGAC-3′ R: 5′-TGCTCCGGCTCAAACCATC-3′	144	NM_012922.2
Caspase-9	F: 5′- GAGGTGAAGAACGACCTGAC-3′ R: 5′-AGAGGATGACCACCACAAAG-3′	103	NM_031632.2
GABA-AR4α	F: 5′-CAGACATATATCCCGTGCATCA-3′ R: 5′-CAGACAGCTATGAACCAATCCA-3′	194	NM_080587.3
NMDA-subunit 1	F: 5′- CCAGTCAAGAAGGTGATCTGCAC -3′ R: 5′- TTCATGGTCCGTGCCAGCTTGA -3′	125	NM_001287423.1
GAPDH	F: 5´-ATGACTCTACCCACGGCAAG-3′ R: 5´-TACTCAGCACCAGCATCACC-3′	136	NM_017008.4

**Figure 3:**
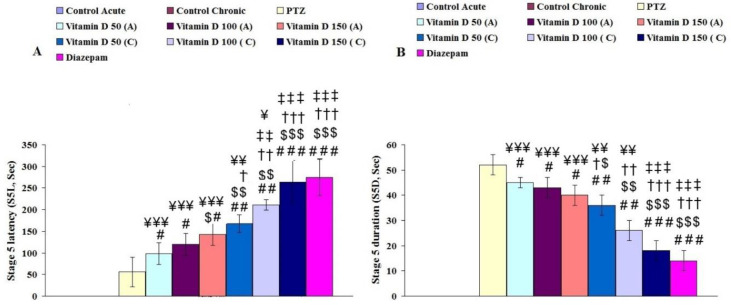
Latency and duration of general tonic and clonic seizures (S5L: A and S5D: B) in different group. A: Acute, C: Chronic. #, ## and ### for *P*<0.05, *P*<0.01 and *P*<0.001, respectively (vs, epileptic group (PTZ)). $, $$, and $$$ for *P*<0.05, *P*<0.01 and *P*<0.001, respectively (vs, Vitamin D 50 acute (A)). †, †† and ††† for *P*<0.05, *P*<0.01 and *P*<0.001, respectively (vs, Vitamin D 100 acute (A)). ‡, ‡‡ and ‡‡‡ for *P*<0.05, *P*<0.01 and *P*<0.001, respectively (vs, Vitamin D 150 acute (A)). ¥, ¥¥ and ¥¥¥ for *P*<0.05, *P*<0.01 and *P*<0.001, respectively (vs, Diazepam). Control: n=10, PTZ: n=10, Diazepam: n=10, Vitamin D 50 acute (A): n=6, Vitamin D 100 acute (A): n=6, Vitamin D 150 acute (A): n=6, Vitamin D 50 chronic (C): n=6, Vitamin D 100 chronic (C): n=6, Vitamin D 150 chronic (C): n=6

**Figure 4:**
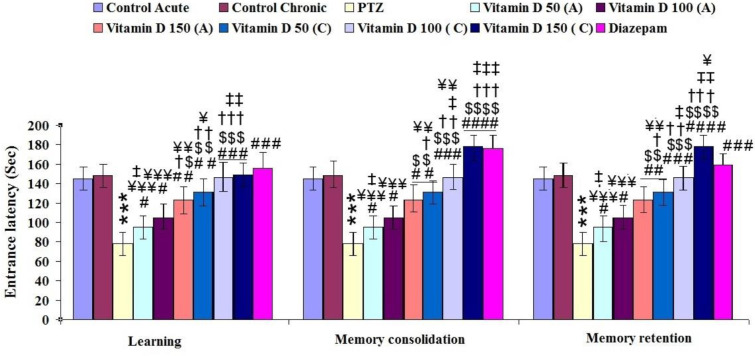
The effect of acute and chronic consumption of different doses of vitamin D on memory and learning in different experimental groups. Results are shown as mean ± SEM. A: Acute, C: Chronic. *** for *P*<0.001 (vs, control group), #, ##, ### and #### for *P*<0.05, *P*<0.01, *P*<0.001 and *P*<0.0001, respectively (vs, epileptic group (PTZ)). $, $$, $$$ and $$$ for *P*<0.05, *P*<0.01, *P*<0.001 and *P*<0.0001, respectively (vs, Vitamin D 50 acute (A)). †, †† and ††† for *P*<0.05, *P*<0.01 and *P*<0.001, respectively (vs, Vitamin D 100 acute (A)). ‡, ‡‡ and ‡‡‡ for *P*<0.05, *P*<0.01 and *P*<0.001, respectively (vs, Vitamin D 150 acute (A)). ¥, ¥¥ and ¥¥¥ for *P*<0.05, *P*<0.01 and *P*<0.001, respectively (vs, Diazepam). Control: n=10, PTZ: n=10, Diazepam: n=10, Vitamin D 50 acute (A): n=6, Vitamin D 100 acute (A): n=6, Vitamin D 150 acute (A): n=6, Vitamin D 50 chronic (C): n=6, Vitamin D 100 chronic (C): n=6, Vitamin D 150 chronic (C): n=6

**Table 2:** The mean± SEM of parathyroid hormone (PTH), vitamin D, calcium and phosphorus in the serum of controlled and treated animals

**Group**	**Control**		**PTZ **	**Diazepam**	**Vitamin D (acute)**			**Vitamin D (chronic)**		
**Subgroup**	Acute (n=5)	Chronic (n=5)	(n=10)	(n=10)	50 (n=6)	100 (n=6)	150 (n=6)	50 (n=6)	100 (n=6)	150 (n=6)
**PTH (**pg/mL)	24.3 ±2	23.7±2	39.3±3 ***	26.3±2 ##	22.1 ±3 ##‡¥	18.3±3###$¥	16.3±1###$¥	17.2±3 ###$¥	21±1.3##‡¥	27.7±3#$†‡‡
**Vitamin D** **(**ng/mL**)**	12.8±2.1	13.1±2	6.6±1 **	12.6±2 ##	6.3±2 †¥‡	8.1±3# $¥	12.7±2##$$†	23.7±2 ###$$$††¥	24.7±3###$$$††¥	27.8±4###$$$††¥¥
**Calcium** **(**mg/dL**)**	8.9±1	9.1±1	13.7±1.3 *	7.7±1 ##	13.2 ±1¥¥	11.7±2 ¥	8.8±3#$	7.7±1.3 ## $	9.1±2#$	8.4±2 #$
**Phosphorus (**mg/dL**)**	5.7±1	6.3±1	13.2±1.3 **	6.8±2 ##	12.1±2 ¥	11.1± 2 ¥	9.3±3 #$¥	4.7±1.3##$$††‡‡	5.9±1##$$††‡‡	6.3±1##$$††‡‡

*, ** and *** for *P*<0.05, *P*<0.01 and *P*<0.001 (vs, control groups), #, ## and ### for *P*<0.05, *P*<0.01 and *P*<0.001, respectively (vs, epileptic group (PTZ)). $, $$, and $$$ for *P*<0.05, *P*<0.01 and *P*<0.001, respectively (vs, Vitamin D 50 acute (A)). † and †† for *P*<0.05 and *P*<0.01, respectively (vs, Vitamin D 100 acute (A)). ‡ and ‡‡ for *P*<0.05, and *P*<0.01, respectively (vs, Vitamin D 150 acute (A)). ¥, and ¥¥ for *P*<0.05 and *P*<0.01, respectively (vs, Diazepam)

**Table 3:** The effect of acute and chronic consumption of different doses of vitamin D on the oxidative and inflammatory status of the brain hippocampus of different experimental groups

Group	Control		PTZ	Diazepam	Vitamin D (acute)			Vitamin D (chronic)		
Subgroup	Acute	Chronic			50	100	150	50	100	150
SOD	13.1±3	13.3±1	4.7±1 *	11.1±1 ###	5.7±1.3 ‡¥	7.3±1 #¥	8.7±1 ##¥ $	8.7±1 ##$	11.2±1 ###$$†‡	12.9±2 ###$$$††‡
CAT	7.6±3	7.8±2	3.7±1 *	6.8±1 #	4.7±1 ¥	5.2±1 #‡¥#	5.7±1 #	7.7±1 ##$†‡	9.7±2 #$$†‡‡¥	11.3±1 ##$$$††‡‡¥
GPX	33.6±7	32.4±3	14.2±2 *	26.4±4 #	15.6±1 ‡¥	17.3±3 #¥	19.7±2 #$¥	23±6 ##$†‡	27.1±2 ##$$††‡	31±3 ¥###$$$††‡‡
GSH	18.6±3	17.6±2	7.5±2 *	16.3±2 #	8.7±1 ¥	9.8±2 #	11.3±3 ##$¥	11.2±2 ##$¥	14.7±1 ##$$	17.7±3 ###$$
MDA	6.6±1	7.2±1	23.8±3 *	13.6±1 #	21.4±3 †¥	18.1±2 #¥	17.1±1 #$¥	16±3 #$	12±3 ##$$†‡	8.1±3 ###$$$††‡‡¥
TNF-α	39.6±2	38.2±2	63.3±1 *	55.3±3 #	61.4±2 ‡ ¥	59.6±2	52.1±2 #$†	51.8±3 #$†	44.5±2 #$††‡¥	36.8±3 ##$$††‡‡¥¥
IL-1β	7.8±2	7.5±2	16.7±2 *	11.4±2 ##	15.2±3 ‡¥	17.8±3 ¥	18.2±2 ¥	15.7±3 ‡¥	12.1±3 #$†‡	9.4±3 ##$$††‡‡

* for *P*<0.05 (vs, control group), #, ## and ### for *P*<0.05, *P*<0.01 and *P*<0.001, respectively (vs, epileptic group (PTZ)). $, $$, and $$$ for *P*<0.05, *P*<0.01 and *P*<0.001, respectively (vs, Vitamin D 50 acute). †, †† and ††† for *P*<0.05, *P*<0.01 and *P*<0.001, respectively (vs, Vitamin D 100 acute). ‡ and ‡‡ for *P*<0.05 and *P*<0.01, respectively (vs, Vitamin D 150 acute). ¥ and ¥¥ for *P*<0.05 and *P*<0.01, respectively (vs, Diazepam)

**Figure 5:**
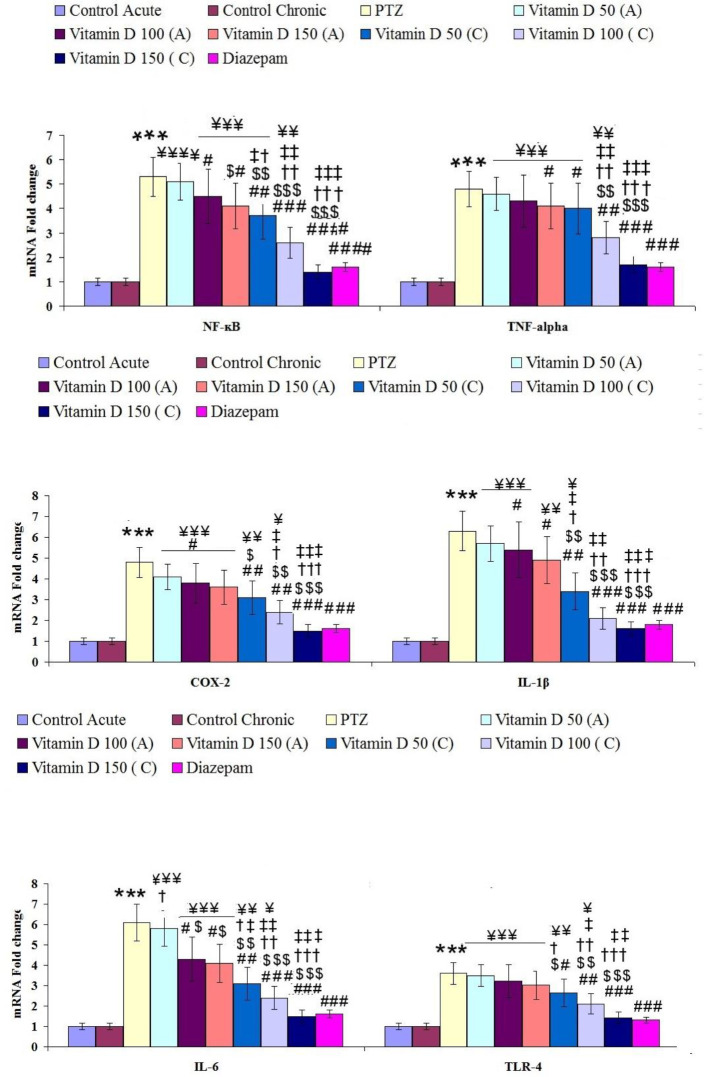
The mean± SEM of expression involved in inflammation in hippocampal tissue of rats in controlled and treated groups. *** for *P*<0.001 (vs, control group), #, ## and ### for *P*<0.05, *P*<0.01 and *P*<0.001, respectively (vs, epileptic group (PTZ)). $, $$, and $$$ for *P*<0.05, *P*<0.01 and *P*<0.001, respectively (vs, Vitamin D 50 acute (A)). †, †† and ††† for *P*<0.05, *P*<0.01 and *P*<0.001, respectively (vs, Vitamin D 100 acute (A)). ‡, ‡‡ and ‡‡‡ for *P*<0.05, *P*<0.01 and *P*<0.001, respectively (vs, Vitamin D 150 acute (A)). ¥, ¥¥ and ¥¥¥ for *P*<0.05, *P*<0.01 and *P*<0.001, respectively (vs, Diazepam). Control: n=10, PTZ: n=10, Diazepam: n=10, Vitamin D 50 acute (A): n=6, Vitamin D 100 acute (A): n=6, Vitamin D 150 acute (A): n=6, Vitamin D 50 chronic (C): n=6, Vitamin D 100 chronic (C): n=6, Vitamin D 150 chronic (C): n=6

**Figure 6:**
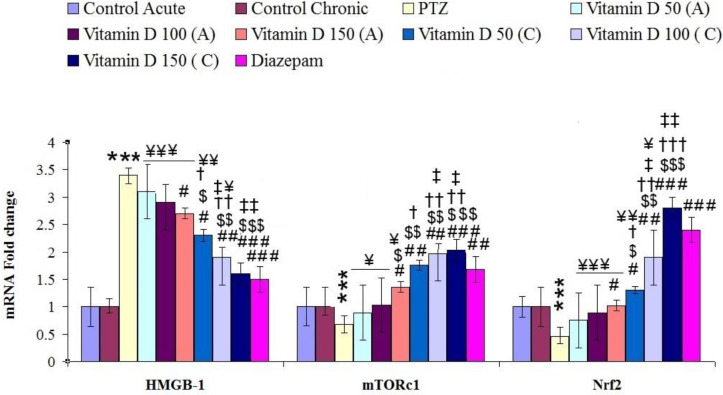
the mean± SEM of HMGB-1, mTORc1 and Nrf-2 expression in hippocampal tissue of brain in controlled and treated groups. *** for *P*<0.001 (vs, control group), #, ## and ### for *P*<0.05, *P*<0.01 and *P*<0.001, respectively (vs, epileptic group (PTZ)). $, $$, and $$$ for *P*<0.05, *P*<0.01 and *P*<0.001, respectively (vs, Vitamin D 50 acute (A)). †, †† and ††† for *P*<0.05, *P*<0.01 and *P*<0.001, respectively (vs, Vitamin D 100 acute (A)). ‡, ‡‡ and ‡‡‡ for *P*<0.05, *P*<0.01 and *P*<0.001, respectively (vs, Vitamin D 150 acute (A)). ¥, ¥¥ and ¥¥¥ for *P*<0.05, *P*<0.01 and *P*<0.001, respectively (vs, Diazepam). Control: n=10, PTZ: n=10, Diazepam: n=10, Vitamin D 50 acute (A): n=6, Vitamin D 100 acute (A): n=6, Vitamin D 150 acute (A): n=6, Vitamin D 50 chronic (C): n=6, Vitamin D 100 chronic (C): n=6, Vitamin D 150 chronic (C): n=6

**Figure 7:**
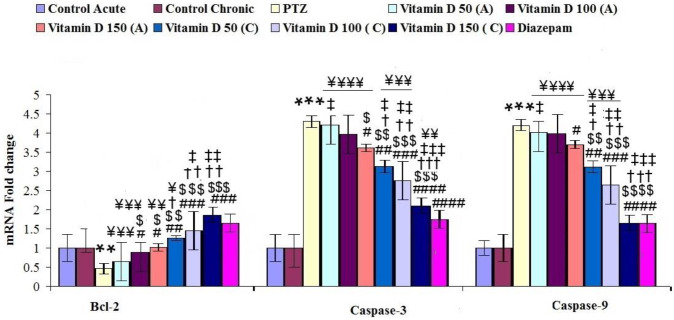
Mean ± SEM expression of genes involved in apoptosis in brain hippocampal tissue in controlled and treated groups *** for *P*<0.001 (vs, control group), #, ##, ### and #### for *P*<0.05, *P*<0.01, *P*<0.001 and *P*<0.0001, respectively (vs, epileptic group (PTZ)). $, $$, and $$$ for *P*<0.05, *P*<0.01 and *P*<0.001, respectively (vs, Vitamin D 50 acute (A)). †, †† and ††† for *P*<0.05, *P*<0.01 and *P*<0.001, respectively (vs, Vitamin D 100 acute (A)). ‡, ‡‡ and ‡‡‡ for *P*<0.05, *P*<0.01 and *P*<0.001, respectively (vs, Vitamin D 150 acute (A)). ¥, ¥¥, ¥¥¥ and ¥¥¥¥ for *P*<0.05, *P*<0.01, *P*<0.001 and *P*<0.0001, respectively (vs, Diazepam). Control: n=10, PTZ: n=10, Diazepam: n=10, Vitamin D 50 acute (A): n=6, Vitamin D 100 acute (A): n=6, Vitamin D 150 acute (A): n=6, Vitamin D 50 chronic (C): n=6, Vitamin D 100 chronic (C): n=6, Vitamin D 150 chronic (C): n=6

**Figure 8:**
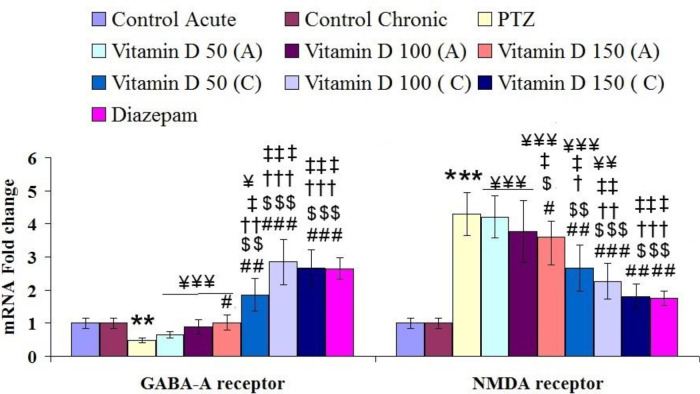
Mean EM SEM expression of GABA-A and NMDA receptors in brain hippocampal tissue in controlled and treated groups.*** for *P*<0.001 (vs, control group), #, ## and ### for *P*<0.05, *P*<0.01 and *P*<0.001, respectively (vs, epileptic group (PTZ)). $, $$, and $$$ for *P*<0.05, *P*<0.01 and *P*<0.001, respectively (vs, Vitamin D 50 acute (A)). †, †† and ††† for *P*<0.05, p<0.01 and *P*<0.001, respectively (vs, Vitamin D 100 acute (A)). ‡, ‡‡ and ‡‡‡ for *P*<0.05, *P*<0.01 and p<0.001, respectively (vs, Vitamin D 150 acute (A)). ¥, ¥¥ and ¥¥¥ for *P*<0.05, *P*<0.01 and *P*<0.001, respectively (vs, Diazepam). Control: n=10, PTZ: n=10, Diazepam: n=10, Vitamin D 50 acute (A): n=6, Vitamin D 100 acute (A): n=6, Vitamin D 150 acute (A): n=6, Vitamin D 50 chronic (C): n=6, Vitamin D 100 chronic (C): n=6, Vitamin D 150 chronic (C): n=6

## Results

***Acute and chronic intake of different doses of vitamin D could improve PTZ-induced seizures.***

Latency of general tonic and clonic seizures (S5L) was significantly lower in the epileptic group than in other groups (F (7, 55): 3.48, *P*≤0.001, Figure 3). This parameter significantly improved in the epileptic groups receiving different doses of vitamin D acutely and chronically compared with the epileptic group (F (7, 55): 6.37, *P*≤0.01, Figure 3A). Duration of general tonic and clonic seizures (S5D) in the epileptic group was significantly higher than that of other groups (F (7, 55): 5.39, *P*≤0.001, Figure 3B). Reversely this parameter decreased by treating acute and chronic different doses of vitamin D (F (7, 55): 2.23, *P*≤0.01, Figure 3B). In addition, a comparison of the results between acute and chronic intake of vitamin D showed that chronic intake of high-dose vitamin D had better therapeutic effects than acute intake of the same dose.

***Acute and chronic intake of different doses of vitamin D could improve cognitive impairment in rats with epilepsy***

A passive avoidance test was performed to measure changes in learning and memory ability after acute and chronic treatment of rats with epilepsy with vitamin D in different doses. This test is based on the animal’s ability to learn and recall the experience of shock and prevent re-entry into the darkroom. Animals with epilepsy took less time to enter the dark chamber than the control group (Figure 4). However, the PTZ-animals treated with vitamin D showed a prolonged latency to enter the dark compartment compared with the epileptic group. In the learning phase, the latency to enter into the dark compartment was lower in the epileptic group than in other groups (F (9, 65): 6.37, *P*≤0.01, Figure 4). Moreover, a significant impairment in both consolidation and memory retention occurred by PTZ injection in the epileptic group (F (9, 65): 3.48, *P*≤0.01, Figure 4). Acute and chronic treatment with different doses of vitamin D could significantly improve the entrance latency, consolidation, and memory retention compared with the epileptic group. The results also showed well that memory and the ability to learn, in epileptic rats in a dose-dependent manner, were significantly improved by acute and chronic intake of vitamin D.

***Acute and chronic intake of different doses of vitamin D could improve changes in PTH, vitamin D, calcium, and phosphorus in the serum of rats with epilepsy***

Changes in PTH, vitamin D, calcium, and phosphorus in the serum of controlled and treated animals are shown in Table 2. Elevated serum PTH level was well observed in rats with epilepsy compared with the control group (F (9, 65): 3.44, *P*≤0.001, Table 2). Serum levels of vitamin D, calcium, and phosphorus were significantly reduced in epileptic rats compared with the control group. However, acute and chronic treatment with different doses of vitamin D could effectively improve changes in serum levels of these parameters in rats with epilepsy (Table 2). 

***Changes in oxidative and inflammatory biomarkers in hippocampal tissues of controlled and treated animals***

The results showed that inflammatory factors and oxidative damage biomarkers significantly altered in the hippocampal tissue in the epileptic group compared with the control group (Table 3). The levels of anti-oxidant enzymes significantly reduced in the epileptic group compared with the control group (Table 3). MDA level and inflammatory factors also significantly increased in the epileptic group compared with the control group. The results also showed that acute and chronic intake of vitamin D in different doses in animals with epilepsy improved oxidative and inflammatory parameters compared with untreated epileptic animals. Comparison of the results in acute and chronic vitamin D groups showed that chronic consumption of vitamin D in a dose-dependent manner improved these parameters (Table 3).

***Chronic and acute intake of vitamin D was able to improve the expression of genes involved in inflammation.***

In this study, the therapeutic acute and chronic effects of different doses of vitamin D on PTZ-induced inflammation were studied by evaluating the expression of HMGB1, NF-ĸB, TNF-α, IL-1β COX-2, and IL-6 genes in hippocampal tissue. Expression of NF-ĸB, TNF-α, IL-1β, COX-2, and IL-6 significantly increased in the hippocampal tissue of epileptic rats compared with the control group (Figure 5). The expression of these genes plays an important role in the development of inflammatory damage caused by repeated injections of PTZ. HMGB1 is used as a biomarker for neuroinflammation, epilepsy, and cognitive impairment. TLR-4 as a receptor for HMGB1 is involved in inflammatory activities. The results showed that the expression of these two genes was significantly increased in the hippocampal tissue of epileptic rats compared with the control group (Figures 5 and 6). While acute and chronic treatment by different doses of vitamin D and diazepam could significantly improve their expression compared with epileptic rats (Figures 5 and 6). 

***Chronic and acute intake of vitamin D could improve the expression of Nrf-2 and mTORc1***

Nrf2 gene expression, which regulates oxidative status, significantly decreased in the hippocampal tissue of epileptic rats compared with the control group (F (9, 65): 2.59, *P*≤0.001, Figure 6). However, acute and chronic treatment with different doses of vitamin D and diazepam could increase its expression and enhance cellular anti-oxidant responses (Table 3, Figure 6). Expression of mTORc1 which regulates many cellular responses and pathways significantly decreased in the hippocampal tissue of epileptic rats compared with the control group. Expression of this gene significantly increased by acute and chronic treatment with different doses of vitamin D and diazepam (Figure 6).

***Chronic and acute intake of vitamin D could improve the expression of genes involved in*** ***apoptosis***

In this study, the expression of genes involved in apoptosis was evaluated in hippocampal tissue of the brain in controlled and treated groups. The expression of the anti-apoptotic gene, Bcl2 in hippocampal tissue was significantly reduced in epileptic rats compared with the control group (F (9, 65): 3.15, *P*≤0.01, Figure 7). However, its expression significantly improved by chronic and acute treatment using different doses of vitamin D and diazepam in treated groups compared with the epileptic group (Figure 7). In addition, the expression of caspase-3 and -9 in this tissue significantly increased in epileptic rats compared with the control group. While acute and chronic treatment with different doses of vitamin D and diazepam significantly reduced their expression in treated groups compared with the epileptic group (Figure 7).

***Chronic and acute intake of vitamin D was able to improve the expression of*** ***GABA-A and NMDA receptors in hippocampal tissue***

The expression of the GABA-A receptor in hippocampal tissue was significantly reduced in epileptic rats compared with the control group (F (9, 65): 3.48, *P*≤0.01, Figure 8). While chronic and acute treatment with different doses of vitamin D could significantly improve its expression in treated groups compared with the epileptic group. In addition, the expression of NMDA receptors in this tissue significantly increased in epileptic rats compared with the control group (F (9, 65): 3.59, *P*≤0.001, Figure 8). While acute and chronic treatment with different doses of vitamin D significantly reduced its expression in treated groups.

## Discussion

Evaluations of S5L and S5D along with shuttle box test results in epileptic animals showed that PTZ injection could successfully induce seizures, epileptic-like behaviors, and cognitive dysfunction. These results indicated that PTZ could significantly cause epilepsy in rats which has been well demonstrated by previous studies (24, 33). In fact, an increase in S5D and a decrease in S5L indicate an increase in excitability of the nerve cells in the hippocampus and cortex due to changes in the function of inhibitory and excitatory pathways. The main mechanism of neuronal excitability is action potential that can result from increased excitatory synaptic neurotransmission (glutamate) or decrease in inhibitory neurotransmission (GABA) by altering the activity of voltage-dependent ion channels in favor of membrane depolarization. The net result of the activity of these two neural transmissions indicates the occurrence of the action potential. 

Studies have shown that PTZ as a GABA-A receptor antagonist suppresses the function of inhibitory synapses and leads to increased neuronal activity (34), which along with other injuries such as apoptosis, inflammation, and oxidative damage can lead to epileptic-like behaviors (35). Yuan *et al*. (2020) showed that PTZ causes inflammation, apoptosis, and oxidative stress in hippocampal tissue. These researchers showed that the levels of GABA, acetylcholinesterase, monoamines, anti-oxidant enzymes, Nrf2, and heme oxygenase-1 (HO-1) decreased and glutamate, pro-inflammatory cytokines and apoptotic agent levels in hippocampal tissue increased in epileptic mice (35). Consistent with these results, our results showed that the activity of anti-oxidant enzymes along with expression of Nrf2 reduced in the hippocampal tissue of epileptic rats. In this regard, events leading to the onset of oxidative damage and apoptosis lead to destruction of nerve cells, which play an important role in causing long-term seizures and disruption of cognitive processes (36).

Our results in line with these studies showed a decrease in expression of Bcl2 and an increase in caspase-3 and -9 in epileptic animals. It is well known that apoptosis and neuronal destruction occur following recurrent epileptic seizures (37) due to a decrease in GABA levels, acetylcholinesterase activity and monoamine content, and an increase in glutamate levels in the hippocampal tissue of epileptic animals (35). Part of the net effect of these processes is to increase the concentration of intracellular calcium, which is associated with the onset of apoptosis and oxidative damage. In addition, astrocytes and microglia increase after seizures in the hippocampus. Meanwhile, active glial cells and necrotic neurons release various inflammatory mediators including IL-1β HMGB1, IL-6, TNF-α, and COX-2 after seizures (10, 38). 

Our results showed that the levels of TNF-α, IL-1β, COX-2, and mTORc1 along with the gene expression of NF-κB/TNF-α/IL-1β, HMGB1/TLR4/IL-1β/NF-κB, and HMGB1/TNF-α pathways increased in hippocampal tissue of epileptic rats. These results indicated an unpleasant condition that not only destroys hippocampal tissue and worsens seizures but also causes cognitive impairments. A situation that in many studies in line with these results has been well established (10, 38). Our results showed that HMGB1 expression increased in hippocampal tissue of epileptic rats. HMGB1 is released by microglia, astrocytes, and necrotic neurons. It binds to multiple receptors such as advanced glycation end products (RAGE) and toll-like receptor (TLR)-4 on the target cells (38). Changes in HMGB1 levels can modulate the severity of acute and chronic seizures through cellular signaling pathways. HMGB1/TLR4/IL-1β/IL-1R pathway, HMGB1/TNF-α/TRADD pathway, and TRAF-2 are the key initiators of neuroinflammation for epilepsy and cognitive impairments (39). The HMGB1/TLR4/IL-1β/IL-1R pathway activates effector molecules including MyD88, IRAC1, and NF-κB and its cellular consequences are a decrease in calcium-activated potassium channels and GABA-A receptors, and an increase in NMDA receptors. HMGB1/TNF-α/TRADD and TRAF-2 pathways activate effector molecules including ASK1, PI3K, AKT, caspase 3, NF-κB, and AP-1, and its cellular consequences are cell death and increased transcription of IL-1, IL-6, and COX-2 (39). A study (2018) showed that the HMGB1/TLR4/NF-κB pathway mediates the activation of microglia in epilepsy (38). In fact, the net result of these changes is excessive irritability and an increase in the duration, frequency of epileptic seizures, and impaired cognitive function (10). The PI3K/Akt/mTOR pathway and caspase 3 are the effector mediators in apoptosis, and NF-κB is the key initiator and the main regulator of inflammation (39). *In vitro* studies and animal models showed that an increase in IL-1β contributes to initiating seizures and epilepsy (40). Its primary source is microglia and is regulated by NF-κB. However, in a feed-forward manner, IL-1β can induce NF-κB signaling via TRAF6, thus forming an auto-regulatory loop (41). A comprehensive study on the role of IL-1β in epilepsy (2007) showed that IL-1β signaling induces neuronal excitability through alterations of ion channels and neurotransmitter receptors, and stimulates an inflammatory cascade through NF-κB (42). IL-1β signaling also activates microglial cells and recruitment of leukocytes across the blood-brain barrier, releasing a wide range of oxidative damage mediators and inflammatory factors including COX-2, IL-6, and TNF-α. In support of these findings, knockdown or inhibition of the IL-1R1 receptor results in decreased production of the downstream cytokines (8). Moreover, a 2017 study showed that IL-1β activates the PI3K/Akt/mTOR pathway in primary hippocampal neurons, enhancing synaptic vesicle endocytosis in hippocampal neurons and increasing synaptophysin (SYN) expression (40). Xiao *et al*. (2016) also showed that mTOR is continuously activated in the rat mesial temporal lobe epilepsy (MTLE) model and children with MTLE and is associated with the IL-1β (11). They also showed in another study that inhibition of PI3K/Akt/mTOR pathway by LY294002 and rapamycin reduced Akt and 70S6K expression and inhibited IL-1β-induced morphological changes in hippocampal neurons (43). Our results in line with these studies showed that mTORc1 expression increased in the hippocampal tissue of epileptic rats, while acute and chronic treatment with vitamin D could reduce its expression. Our results and those of others reported well that NF-κB is increased in the hippocampal tissue of epileptic animals (44). Increases in levels of intracellular Ca^2+^ and ROS such as H_2_O_2_ can be potent activators of NF-κB/TNF-α (45). Our results also showed that expression of COX-2 in the hippocampal tissue of epileptic rats increases which is consistent with the results of previous studies. COX-2 is a component of the inflammatory system in disorders of the nervous system, including epilepsy. In the last decade, COX-2-mediated neuritis and its associated pathways have been considered as potential therapeutic targets for improvement of epilepsy (10). HMGB1 and increased intracellular Ca^2+^ concentrations, phenomena that are the major causes of neuronal hyperexcitability and other cellular damages to neurons and glial cells in epilepsy, are the main stimuli for the expression of COX-2 (10). It is well known that controlling these events is one of the treatment options for seizures and epilepsy.

The results of this study showed that the evaluated seizure responses along with inflammation, oxidative damage, apoptosis, and cognitive behaviors (memory and learning) were effectively improved in epileptic rats with acute and chronic treatment with different doses of vitamin D. They also showed well that acute and chronic treatment of epileptic rats with different doses of vitamin D3 improved cognitive function and seizure responses induced by PTZ. Our results showed that vitamin D3 supplementation for two weeks delayed the onset (S5L) and reduced the duration of general tonic and clonic seizures (S5D) resulting from PTZ. Animal and human studies have shown that the use of vitamin D reduces the seizure threshold and the frequency of epileptic seizures(23). A study (2012) showed that administration of vitamin D3 in 13 patients with pharmacoresistant epilepsy significantly reduced the number of seizures over a 90-day period (21). Al-Khalifah *et al*. (2018) showed that vitamin D deficiency is highly prevalent among children with epilepsy. They did a study to determine the efficacy of different doses used in the pediatric practice to maintain optimal 25 (OH) vitamin D levels in children with epilepsy and showed that maintaining serum levels of 25 (OH) vitamin D helps control seizures in children with epilepsy (46). Animal studies also showed that vitamin D3 raises the threshold of seizure and reduces its deleterious effect (23). In support of these results, a study (2006) showed that administration of PTZ to mice with a deficiency in vitamin D receptors induces less latency to the seizure beginning and increases seizure susceptibility (47). A review study showed in detail that the use of vitamin D3 could be involved in the improvement of patients with epilepsy by increasing seizure threshold and reducing the number of seizures. Vitamin D could improve these symptoms through genomic and non-genomic roles (48, 49). It has been also demonstrated that vitamin D receptors increased in the hippocampus after pilocarpine-induced seizures (50). The results of these studies in line with our results showed that vitamin D could effectively increase S5L and reduce S5D. Reducing the duration of seizures and increasing the latency time in addition to inhibiting excitatory mechanisms and activating inhibitory mechanisms indicate an improvement in cell-damaging conditions such as oxidative damage, inflammation, and apoptosis in the hippocampus of epileptic rats. The results of our study, in line with the results of previous studies, showed that vitamin D could control PTZ-induced epilepsy well by improving inflammation, oxidative damage, and apoptosis. Moreover, our results showed that the ability to learn, memory consolidation, and memory retention in rats with epilepsy improved after acute and chronic treatment with different doses of vitamin D. The results of our study showed that vitamin D could inhibit NF-κB/TNF-α/IL-1β, HMGB1/TLR4/NF-κB, HMGB1/TLR4/IL-1β, and HMGB1/TNF-α pathways and down-regulated the expression of COX-2 and mTOR in hippocampal tissue of epileptic rats. TLR4 is the main receptor of HMGB1 and is an important immune pattern recognition receptor that controls innate and adaptive immune responses and plays an important role in initiating and regulating inflammation. NF-κB, IL-1β, and TNF-α are located on the downstream TLR4 signaling pathway. Therefore, HMGB1/TLR4/NF-κB/IL-1β and HMGB1/TLR4/NF-κB/TNF-α signaling pathways are the important part of immunoregulatory and neuroinflammation processes. Rearrangement and intervention of any of these signaling pathways affect the occurrence and development of epilepsy. Sadeghi *et al*. (2006) found that vitamin D3 could down-regulate the expression of TLRs in the monocytes and reduce the production of pro-inflammatory cytokines such as TNF-α (51). A 2017 study reported that vitamin D3 effectively reduced the oxidative stress and inflammatory response of adipose tissue in a rat model of high fat diet-induced obesity (52). Zhang *et al*. (2018) indicated that vitamin D effectively reduced inflammatory response and apoptosis in the lung tissue of a mouse model of asthma (53). Another study (2017) also showed that pretreatment with vitamin D3 and its synthetic analogs markedly down-regulated the expression of TLR4, HMGB1, TNF-α, and NF-ĸB (54). The findings of these studies showed well that vitamin D3 has the ability to effectively inhibit the HMGB1/TLR4/ NF-ĸB pathway and can improve the seizure responses. Our results also showed that vitamin D3 could inhibit mTORc1 expression. Previous studies in line with these results have showed that vitamin D3 could inhibit mTORc1 expression and its activity (55, 56). Vitamin D3 inhibits the mTOR signaling pathway by stimulating the expression of DNA damage-inducible transcript 4 (DDIT4), a potent suppressor of mTOR activity (56). DDIT4 mRNA and protein are up-regulated by 1,25(OH)2D treatment resulting in reduced phosphorylation of mTORc1 and decreased cell proliferation and growth (55). In addition, vitamin D3 inhibited the phosphorylation of AKT and mTOR, and downstream targets eukaryotic translation imitation factor 4Ebinding protein 1 and ribosomal protein S6 kinase β-1 (55). These results showed that vitamin D3 could inhibit the PI3K/Akt/mTOR pathway. Our results, consistent with previous results (56), showed that mTOR expression markedly decreased with chronic and acute treatment with different doses of vitamin D3. In this study, in addition to different doses of vitamin D, we evaluated its acute and chronic use in similar doses. Comparison between chronic outcomes and acute outcomes of vitamin D3 showed that chronic ingestion at the same dose produced a more significant response than acute ingestion. This difference in effect is probably due to the activation of processes related to the genetic and non-genetic effects of this vitamin (48, 49). It was reported that genomic and non-genomic pathways associated with vitamin D receptors have a significant role in modulating the responses resulting from the dysfunction of hippocampal neurons (23, 48, 49). In support of these results, studies have shown that vitamin D could play an important role in controlling neural activity by modulating intracellular pathways and is useful in improving patients with neurological diseases such as Alzheimer’s, Parkinson’s, and multiple sclerosis (20). It seems that acute consumption of different doses of this vitamin has only been able to activate non-genetic effects (48). Non-genomic actions of vitamin D involve a wide range of different cell signalings (12, 48, 49) which include 1) activation of signaling molecules, such as phospholipase C and phospholipase A2 (PLA2) and phosphatidylinositol-3 kinase (PI3K), 2) rapid generation of second messengers (Ca^2+^, cyclic AMP, fatty acids, and 3-phosphoinositides such as phosphatidylinositol 3,4,5 trisphosphate), 3) activation of protein kinases, such as protein kinase A, mitogen-activated protein (MAP) kinases, protein kinase C (PKC), and Ca^2+^-calmodulin kinase II, and 4) opening of Ca^2+^ and Cl^-^ channels. The presence of specific receptors and enzymes for vitamin D3 in nerve and glial cells throughout the brain, spinal cord, and peripheral nervous system supports the neurological role of vitamin D (57). This vitamin is well known as a neurosteroid hormone that regulates neurotransmitters and neurotrophins (12). In addition, vitamin D3 is involved in neuroprotection, proliferation, and differentiation of brain cells and brain growth (58). In fact, these vital roles of vitamin D can well support the results of this study and represent a good option for prevention and recovery in patients with epilepsy. In this study, we also showed that acute and chronic use of different doses of vitamin D3 could effectively improve PTZ-induced cognitive impairment in epileptic rats. This effect is well illustrated in various animal models. In addition, many studies believe that vitamin D deficiency is associated with cognitive impairment and Alzheimer’s. It is possible that the improvement in cognitive responses in rats with epilepsy when receiving vitamin D may be due to the anti-inflammatory and anti-oxidant properties of this vitamin. In addition, many studies have shown that this vitamin is involved in the protection and development of the nerve. Our results showed well that in a dose-dependent response, vitamin D could well inhibit inflammatory pathways (HMGB1/TLR4/ NF-ĸB, HMGB1/TLR4/IL-1β, and HMGB1/TNF-α) and oxidative damage (Nrf-2/Enzymes anti-oxidants) and reverse these effects. Improving this condition can play an important role in improving cognitive function. In this study, in order to maintain proper serum concentrations of vitamin D, we prescribed different concentrations of it acutely and chronically. Moreover, serum levels of vitamin D3 were assessed two weeks after starting treatment to determine the successful normalization of vitamin D3 treatment and to rule out possible toxicity. In this study, we also assessed the serum level of PTH, Ca, and P. The results in line with previous studies (46) showed that the level of vitamin D, Ca and P decreased and serum PTH increased in animals with epilepsy. An increase in PTH level probably indicates a secondary response to low levels of serum vitamin D and ionized calcium. It should be noted that although many animal studies support the use of vitamin D to improve the symptoms of epilepsy and inhibit the onset of seizures and its progression in different models, the use of this vitamin in human studies due to toxic effects and confounding factors are limited (23). Therefore, conclusions from clinical studies are somewhat difficult. There is a critical need for larger clinical trials to establish the safety and efficacy of vitamin D3 in epilepsy. 

The results of this study showed that the expression of the GABA-A receptor decreased and the NMDA receptor subunit increased in the hippocampal tissue of epileptic rats. Consistent with the results of our study, many researchers have reported that in long-term seizures, the number of active GABA-A receptors in the postsynaptic membrane decreased, and the number and activity of NMDA receptors increased. Therefore, this process increased the ability of the nervous system to become overly irritable, which in turn causes convulsive activity. Our results showed well that chronic intake of different doses of vitamin D could effectively increase the expression of the GABA receptor and decrease the NMDA receptor. In addition, it is possible that the improvement in inflammation and oxidative damage has caused glial cells to perform their role better and clear glutamate from the space between the synapses (36, 59). Another important point is the propagation of seizures. Seizure propagation, the process by which a partial seizure develops in the brain, occurs when there is not enough activity to absorb the neurotransmitters released into the synaptic cleft. This leads to a loss of surround inhibition and spread of seizure activity into contiguous areas via local cortical connections, and to more distant areas via long association pathways such as the corpus callosum. This is typically prevented by neuronal hyperpolarization and or by inhibitory neurons. Glutamate and GABA in epilepsy both require active reuptake to be cleared from the synaptic cleft. Transporters for glutamate and GABA exist on neurons and glia (primarily astrocytes) (59). In this case, improving the function of glutamate transporters and inhibiting GABA transporters cause antiepileptic activity. It is possible that vitamin D was involved in this process and was able to prevent the propagation of action potential to nearby neurons, reducing the duration of the seizure period and increasing its delay in onset. However, further studies are needed to elucidate the role of vitamin D in improving the function of these transporters. Vitamin D may play a protective role in improving the prevention of oxidative damage and inflammation, as well as improving cellular signaling pathways. Electrophysiological studies have shown well that vitamin D can have modulatory effects on the activity of hippocampal neurons in the model of animal epilepsy by improving GABAergic pathways and inhibiting glutaminergic pathways (60). 

## Conclusion

It can be concluded that in addition to improving cognitive function in rats with epilepsy, delayed onset of seizure responses (S5L) and shortening of seizure period (S5D) in vitamin D groups may be due to inhibition of inflammatory pathways, oxidative damage, and cellular signaling pathways. Moreover, we have shown that chronic consumption of different doses of vitamin D decreases the expression of NMDA receptors and increases GABA receptors. In fact, vitamin D has been involved in neurotransmission not only through its protective role in inhibiting oxidative damage and inflammation but also through cellular signaling pathways such as the m-TOR pathway. These results clearly showed that taking the proper dose of vitamin D can be a good treatment option for people with epilepsy. However, obtaining the appropriate dose at different ages and disease conditions requires further studies or the use of lower-risk analogs.

## Authors’ Contributions

HJ and SZ Helped with study conception and design; HJ and SZ Performed data analysis and draft manuscript preparation; HJ and SZ Critically revised the paper; SZ Supervised the research; SZ and HJ Approved the final version to be published.

## Conflicts of Interest

The authors declare that no conflict of interest exists.
